# The Effects of Sodium-Glucose Co-transporter 2 Inhibitors on Cardiovascular Outcomes in Heart Failure: A Systematic Review and Meta-Analysis

**DOI:** 10.7759/cureus.98140

**Published:** 2025-11-30

**Authors:** Akshay Maharaj, Sajay Bidhesi, Diya Grover, Parvati Raghunath, Rajiv N Lutchmedial, Adam S Khan, Matthew A Maharaj, Priya V Heera, Ravi Maharaj, Christian Sookhan, Chelsea D Kanhai, Camisha Marshall, Anyse A Calder

**Affiliations:** 1 Internal Medicine, Port of Spain General Hospital, Port of Spain, TTO; 2 Internal Medicine, San Fernando General Hosptial, Couva, TTO; 3 Internal Medicine, Mount Sinai Hospital, Chicago, USA; 4 Surgery, Medical Associates Hospital, St. Joseph, TTO; 5 Internal Medicine, University of the West Indies Eric Williams Medical Sciences Complex, St. Augustine, St. Augustine, TTO; 6 General Practice, Sangre Grande General Hospital, Sangre Grande, TTO; 7 Accident and Emergency, North Central Regional Health Authority, Mt. Hope., St. Joseph, TTO; 8 Medical Sciences, University of the West Indies Eric Williams Medical Sciences Complex, St. Augustine, St Augustine, TTO; 9 Surgery, University of the West Indies Eric Williams Medical Sciences Complex, St. Augustine, St. Augustine, TTO; 10 Emergency Medicine, North Central Regional Health Authority, St. Augustine, TTO; 11 Emergency Medicine, Port of Spain General Hospital, Port of Spain, TTO

**Keywords:** cardiology research, cardiovascular disease, dapagliflozin, empagliflozin, heart failure, sodium-glucose cotransporter 2 inhibitor, systematic review and meta analysis

## Abstract

Sodium-glucose co-transporter 2 (SGLT2) inhibitors, initially developed to treat type 2 diabetes mellitus, work by blocking SGLT2 proteins in the proximal convoluted tubules of the nephrons, thereby preventing glucose reabsorption into the bloodstream. Subsequently, its mechanism reveals additional cardiovascular and renal benefits, necessitating further investigation into its effects on heart failure. This condition affects millions and leads to diminished quality of life, frequent hospitalizations, and high mortality rates. This review aims to evaluate several large randomized controlled trials (RCTs) investigating the favourable outcomes of SGLT2 inhibitors on heart failure and its related variables, including hospitalizations secondary to the condition, mortality rate, kidney function, and left ventricular ejection fraction (LVEF). Nine RCTs were reviewed systematically and meta-analyzed to assess the effectiveness of SGLT2 inhibitors. A pooled risk ratio was calculated under a random-effects model, along with heterogeneity analysis, to summarize the overall effect across studies. The studies analyzed two primary clinical outcomes of mortality and rehospitalization due to heart failure. SGLT2 inhibitors were associated with a 20% relative reduction in mortality (risk ratio (RR) = 0.80) and a 24% relative risk reduction of rehospitalization due to heart failure (RR = 0.76). These findings support the cardioprotective effects of SGLT2 inhibitors, as well as significant reductions in hospitalization and mortality. The efficacy of SGLT2 inhibitors was observed across diverse demographic and clinical subgroups, supporting their role as a valuable therapeutic option for managing heart failure.

## Introduction and background

Heart failure (HF) occurs when the heart cannot pump blood effectively, leading to reduced blood flow to organs and tissues. It affects over 64 million people worldwide and poses a growing public health burden, driven by aging populations and improved survival from other cardiovascular conditions [1]. HF also places a significant strain on healthcare systems through frequent hospitalizations, intensive pharmacologic management, and device-based therapies. For example, in the United States, nearly 20% of patients are rehospitalized within 30 days of an HF-related admission. From a patient perspective, HF severely impairs quality of life, with symptoms such as shortness of breath, fatigue, and limited physical activity, which are associated with increased disability and high mortality risk [2].

Improving clinical outcomes in HF, specifically reducing cardiovascular death and HF-related hospitalizations, remains a central goal. Although interventions have improved survival, data on the consistency of benefit across diverse HF populations and the real-world impact on rehospitalization rates are still limited. Outcomes such as left ventricular ejection fraction (LVEF) and renal function are also important but are inconsistently reported across trials, leaving gaps in evidence that may inform clinical practice.

Sodium-glucose co-transporter 2 (SGLT2) inhibitors block glucose reabsorption in the proximal renal tubules, promoting glycosuria, osmotic diuresis, and natriuresis. These changes not only lower blood glucose but also reduce fluid overload and improve cardiac hemodynamics, providing cardiovascular and renal benefits independent of glycemic control [3].

Initially approved for type 2 diabetes mellitus, SGLT2 inhibitors were subsequently found to reduce HF hospitalizations and cardiovascular death, prompting investigation into their use in patients with HF and chronic kidney disease. Several large randomized controlled trials have confirmed these benefits.

The DAPA-HF (Dapagliflozin and Prevention of Adverse Outcomes in Heart Failure) trial demonstrated that dapagliflozin significantly reduced cardiovascular death and HF hospitalization in patients with HF with reduced ejection fraction (HFrEF), regardless of diabetes status [4]. The EMPEROR-Reduced (EMPagliflozin outcomE tRial in Patients With chrOnic heaRt Failure With Reduced Ejection Fraction) trial reported similar benefits with empagliflozin in HFrEF patients. The EMPEROR-Preserved trial extended these findings to patients with HF with preserved ejection fraction (HFpEF), demonstrating effectiveness across HF phenotypes [5]. These trials highlight the utility of SGLT2 inhibitors in a broad spectrum of HF patients, including those with diabetes and varying ejection fraction categories, as well as patients with concomitant renal impairment.

Given the rapid accumulation of evidence, a meta-analysis is necessary to synthesize data from multiple trials, providing a quantitative estimate of the impact of SGLT2 inhibitors on cardiovascular death and HF-related hospitalizations. By addressing gaps in outcomes reporting and examining consistency across diverse populations, this meta-analysis aims to inform clinical decision-making, optimize treatment strategies, and support resource allocation in HF care.

## Review

Method

This study was conducted in accordance with the standardized guidelines for systematic reviews and meta-analyses, utilizing the Preferred Reporting Items for Systematic Reviews and Meta-Analyses (PRISMA) checklist.

Search Strategy

A literature search was conducted across three major databases, including PubMed, the Cochrane Library, and ClinicalTrials.gov, all of which are maintained by the National Library of Medicine (NLM). Pre-defined search terms were used, including “Heart Failure” (including “Chronic Heart Failure”, “Congestive Heart Failure”, “HFrEF”, and “HFpEF”). Outcome-related terminology, such as “LVEF”, “cardiovascular death”, and “HF hospitalization”, was also applied to further refine the literature search, in which the last search was conducted on May 5, 2025.

Eligibility Criteria

Studies included in this analysis were randomized controlled trials (RCTs) that compared the effect of SGLT2 inhibitors (such as empagliflozin, dapagliflozin, or canagliflozin) against a placebo using specific clinical outcomes of interest. These outcomes include cardiovascular death, HF hospitalizations, LVEF, and serum creatinine (SCreat). The included studies must have at least one of these outcomes and a minimum follow-up period of three months from the index day. Study populations must consist of patients with HF (regardless of the type of HF) who are at least 18 years of age. Studies that did not meet these criteria or did not provide full access were excluded from the analysis. Additional exclusion criteria for the analysis included case reports, meta-analyses, systematic reviews, conference abstracts, non-English articles, and studies involving non-human subjects. 

Screening

Three teams of two investigators screened the studies using the aforementioned inclusion and exclusion criteria. The team utilized Zotero to eliminate duplicates. Risk ratios (RRs) and confidence intervals (CIs) for HF hospitalization and death were input into the JASP (version 0.19.0, 2024, JASP Team, University of Amsterdam, Amsterdam, Netherlands) using a random effects model (to account for variability between studies) and created forest plot using measures of heterogeneity with a 95% CI. Chi (Cochran’s Q test and its corresponding p value), and degrees of freedom (df) were interpreted together to determine heterogeneity if Chi > df. I² was used to quantify heterogeneity. If I²>50%, further subgroup analyses would isolate the source of heterogeneity (however, no significant heterogeneity was detected). Studies with incomplete or missing outcome data were excluded from the meta-analysis. This approach ensured that our pooled estimates reflect the most reliable and complete data available across the included studies.

*Risk of Bias Assessment* Risk of Bias is defined as the possibility that the design, conduct, analysis, or reporting of a study has introduced systematic errors that distort the true effect or outcome being measured. The Robvis risk-of-bias domains were used to analyze the bias categories of each study. Robs 2 classified each study into low, high, and some concern risk of bias. These results were obtained after each study was assessed with respect to domains including the randomization process, deviation from intended intervention, missing outcome data, measurement of the outcomes, and the selection of the reported results. The quality of evidence extracted by two independent investigators, with subsequent discrepancy analysis, was employed. A third investigator then settled this through logical reasoning between the two independent investigators.

Results

**Figure 1:**
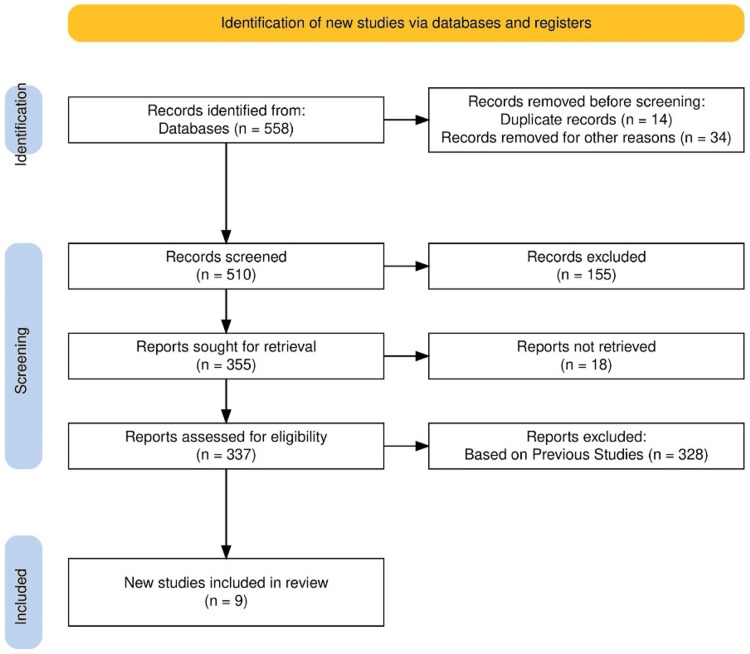
PRISMA flow chart PRISMa: Preferred Reporting Items for Systematic Reviews and Meta-Analyses

A total of 558 articles were screened, which resulted in nine studies that evaluated the effects of SGLT2 inhibitors on two key clinical outcomes of all-cause mortality and HF rehospitalization. The other outcomes of interest, LVEF and SCreat, did not yield enough data to perform a meta-analysis. The study selection process is given in Figure 1. Summary of the studies are given in Table 1.

**Table 1:** Key details of studies included in the review LVEF: left ventricular ejection fraction; CV: cardiovascular; HHF: hospitalization for heart failure; HR: hazard ratio; WR: stratified win ratio

Author(s), Year	Location	Sample size	Intervention	Outcome Measures	Results (Compared to placebo
McMurray et al., 2019 [4]	Multicenter	4744	Dapagliflozin	CV death or HHF	HR 0.74 (95%CI, 0.65, 0.85)
Anker et al., 2021 [5]	Multicenter	5988	Empagliflozin	CV death or HHF	HR 0.79 (95%CI, 0.69, 0.90)
Packer et al., 2020 [6]	Multicenter	3730	Empagliflozin	CV death or HHF	HR 0.70 (95%CI, 0.58, 0.85)
Abdullaev et al., 2022 [7]	Tashkent, Uzbekistan	225	Dapagliflozin	CV death or HHF	HR 0.74 (95%CI, 0.65, 0.85)
Solomon et al., 2022 [8]	Multicenter	6263	Dapagliflozin	CV death or HHF	HR 0.82 (95%CI, 0.73, 0.92
Santos-Gallego et al., 2021 [9]	United States	84	Empagliflozin	Change in LVEF, mean±SD	6.0 ± 4.2
James et al., 2024 [10]	Sweden and United Kingdom	4017	Dapagliflozin	CV death or HHF	HR: 0.95 (95%CI, 0.64, 1.40)
Butler et al., 2024 [11]	Multicenter	6522	Empagliflozin	HHF	HR 0.77 (95%CI, 0.60, 0.98)
Voors et al., 2022 [12]	Multicenter	530	Empagliflozin	Death, HFE, or time to first HFE	WR 1.36 (95%CI, 1.09, 1.68)

**Figure 2:**
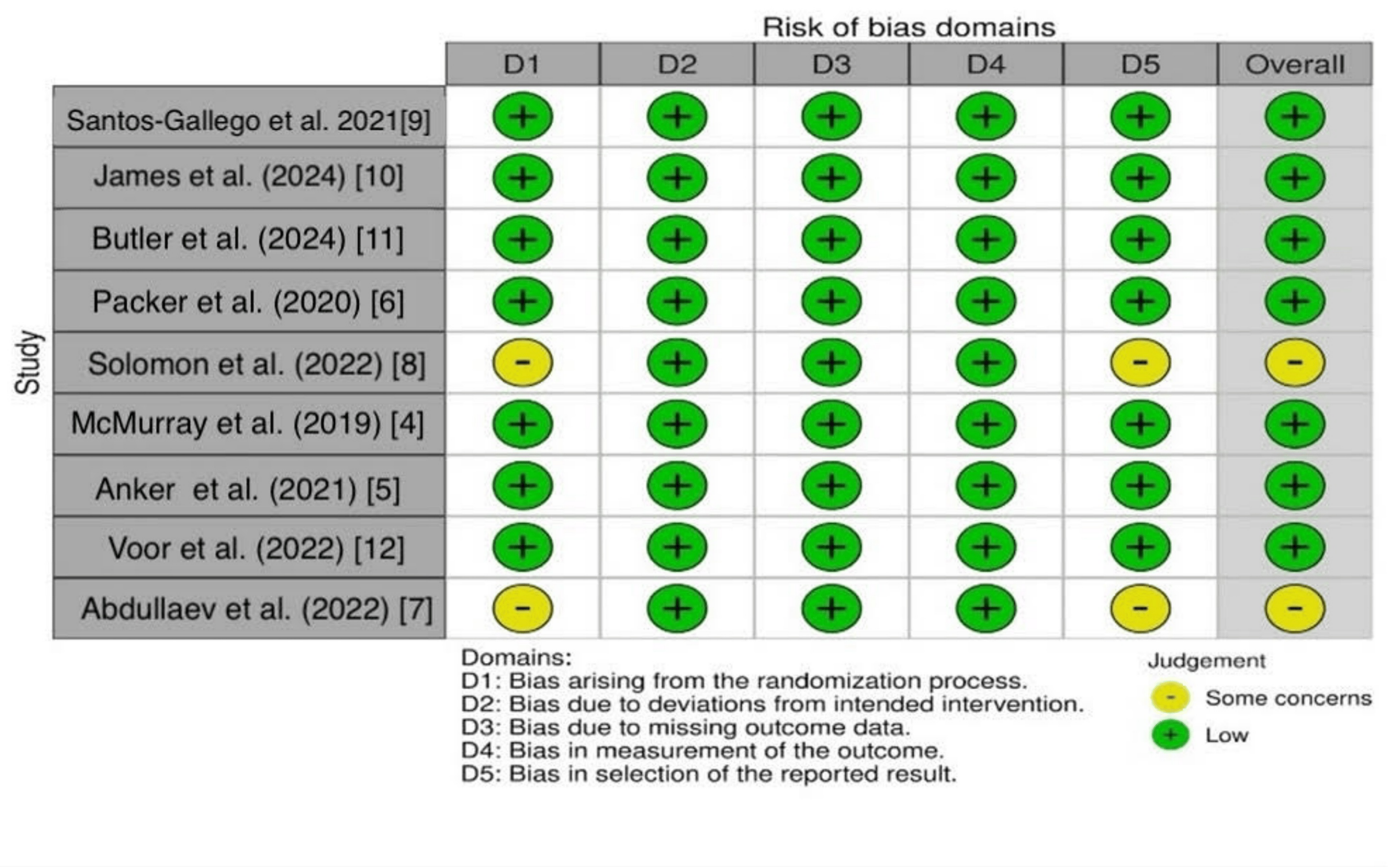
The risk-of-bias traffic light plot The traffic light plot visually summarizes the risk of bias for each included study across key methodological domains (such as random sequence generation, allocation concealment, blinding, incomplete outcome data, selective reporting, and other biases) based on the Cochrane risk of bias tool. Each domain is color-coded: green indicates low risk of bias, yellow indicates unclear risk, and red indicates high risk. References: [4-12]

None of the studies for the traffic light plot met the high risk of bias category. However, two studies noted concerns with the D1 and D5 domains [7,8]. This is due to the absence of blinding, meaning both the investigators and the patients were aware of the interventions, which inherently introduces bias. Additionally, neither study reported the entirety of the results, which led to some concerns. While this could have affected the results of these studies, this was not to an alarming degree; hence, there was only some concern. Otherwise, all of the other studies had a low risk of bias (Figure 2).

**Figure 3:**
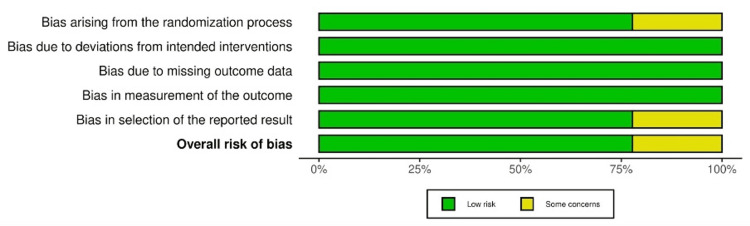
Risk-of-bias bar graph

The bar graph in Figure 3 illustrates that the included studies generally had a low risk of bias, and those that did not were only of some concern. The areas of concern were the randomisation process and the reported results. This was heavily due to the absence of blinding in those research studies.

CVS Death

**Figure 4:**
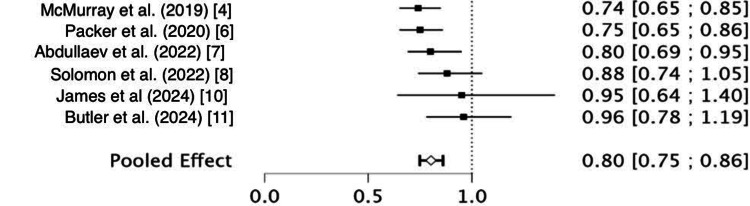
Cardiovascular death forest plot References: [4,6-8,10,11]

The pooled RR for CVS death among patients treated with SGLT2 inhibitors was 0.80 (95%CI: 0.75 to 0.86), indicating a 20% relative reduction in the risk of death compared to control. Individual study estimates consistently favored the intervention, although some had confidence intervals that crossed the null (Figure 4). Heterogeneity analysis revealed a Q statistic of 7.06 with 5 degrees of freedom (df) and a p-value of 0.232, suggesting no statistically significant heterogeneity. The I² value was 29.2%, reflecting low to moderate heterogeneity, and the estimated between-study variance (Tau²) was 0.0029. These results support the validity of pooling the studies under a random-effects model.

HF Rehospitalization

**Figure 5:**
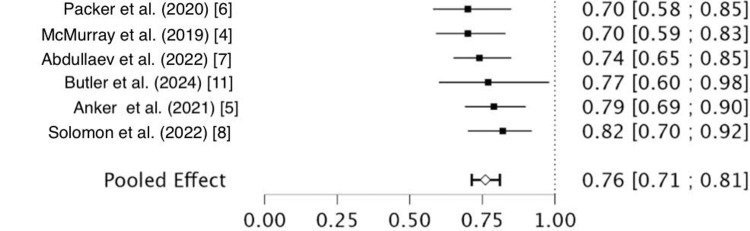
Heart failure rehospitalization forest plot References: [4-8,11]

The pooled RR for HF rehospitalization was 0.76 (95% CI: 0.71 to 0.81), indicating a 24% relative reduction in the risk of rehospitalization with SGLT2 inhibitors. The benefit was consistent across all six studies (Figure 5). There was no evidence of heterogeneity among these studies, as indicated by a Q statistic of 3.28 (df = 5), p-value = 0.657, and an I² of 0%. The between-study variance (Tau²) was also 0, reflecting highly consistent results.

Discussion 

This meta-analysis provides robust evidence that SGLT2 inhibitors significantly improve clinical outcomes in patients with HF. Specifically, treatment with SGLT2 inhibitors was associated with a 20% relative reduction in all-cause mortality (RR = 0.80; 95%CI: 0.75-0.86) and a 24% relative reduction in HF-related rehospitalizations (RR = 0.76; 95% CI: 0.71-0.81). These findings underscore the growing role of SGLT2 inhibitors as a foundational therapy in the management of HF, extending beyond their initial use for glycemic control in type 2 diabetes mellitus.

The mortality benefit observed was consistent across trials, with low to moderate heterogeneity (I² = 29.2%) and no statistically significant between-study variability (p = 0.232). The results suggest that the observed effect is statistically sound and clinically meaningful across diverse patient populations and trial designs. Similarly, the impact on rehospitalization for HF was strikingly consistent, with negligible heterogeneity (I² = 0%, p = 0.657), further reinforcing the reproducibility of the benefit.

These results align with landmark trials, such as EMPA-REG OUTCOME (Empagliflozin Major Prognostic Assessment to evaluate the Reducing Eventa- Glucose OUTcome in Cardiovascular Outcomes and other Major Events) [6], DAPA-HF [7,8], EMPEROR-Reduced [9,10], and DELIVER (Dapagliflozin Evaluation to Improve the Lives of Patients With Preserved Ejection Fraction Heart Failure) [11], which collectively support the cardiovascular benefits of SGLT2 inhibitors in both diabetic and non-diabetic populations. The consistency of benefits across these heterogeneous trials suggests mechanisms of action beyond glucose lowering, including natriuresis, reduction in preload and afterload, improvement in ventricular loading conditions, and favorable modulation of myocardial energetics.

Notably, the trials included in this analysis encompassed a range of patients with preserved and reduced LVEF, suggesting broad efficacy across the spectrum of HF phenotypes. The aforementioned is particularly relevant given the limited therapeutic options previously available for HFpEF.

Despite these strengths, several limitations should be acknowledged. The meta-analysis included only six RCTs, which, although extensive and of high quality, may not fully capture the variability in clinical practice settings. Differences in trial design, follow-up duration, baseline patient characteristics (e.g., New York Heart Association (NYHA) class, renal function, diabetic status), and definitions of HF hospitalization may introduce clinical heterogeneity that is not statistically detectable. Moreover, the potential for publication bias cannot be excluded, although the consistency of findings makes this less likely. As such, it is of most importance to consider studies with SGLT2 use in patients with chronic kidney disease and HF. Findings from studies evaluating the effects of SGLT2 (dapagliflozin [13] and empagliflozin [14]) inhibitors on chronic kidney disease and cardiovascular outcomes consistently demonstrate a reduced mortality risk among patients receiving SGLT2 inhibitors compared with placebo. (from 3.8% to 5.3% risk reductions) These findings were reflective of the results of the meta-analysis, showing consistency from multiple avenues of research, hence improving the validity of the results. Additionally, recent real-world data further reinforce the cardiovascular benefits of SGLT2 inhibitors observed in RCTs. A 2024 nationwide Danish linked-registry cohort study evaluated patients with HFrEF treated with SGLT2 inhibitors such as dapagliflozin and empagliflozin in routine clinical practice [15]. The investigators reported a significant reduction in all-cause and cardiovascular mortality among SGLT2 inhibitor users compared to non-users. Importantly, the Danish study included a broader, less-selected patient population, meaning older individuals with multiple comorbidities, thereby reflecting everyday clinical settings rather than the controlled conditions of RCTs. The consistency of outcomes between this large real-world analysis and prior randomized trials strengthens the external validity of SGLT2 inhibitor therapy in HFrEF and underscores its effectiveness and safety across diverse patient populations.

Ultimately, the current analysis reinforces the cardioprotective role of SGLT2 inhibitors and their clear benefit in reducing both mortality and recurrent hospitalizations in HF patients. Given their favorable safety profile, ease of administration, and efficacy across diverse populations, SGLT2 inhibitors should be considered a key component of guideline-directed medical therapy for HF, irrespective of diabetic status. Future research should continue to explore their role in earlier stages of HF and in combination with other HF therapies and their effect on patients with HF and kidney disease.

## Conclusions

This meta-analysis confirms that SGLT2 inhibitors significantly reduce HF hospitalizations and cardiovascular mortality, reinforcing their established role beyond glycemic control. While previous landmark trials demonstrated these benefits, our analysis highlights their consistent effectiveness across diverse patient populations and study designs, with low heterogeneity supporting generalizability. Importantly, the limited number of studies reporting LVEF and renal outcomes identifies gaps in the current evidence base, suggesting areas for future research. Clinically, these findings support the broad integration of SGLT2 inhibitors into HF management, including in populations underrepresented in earlier trials, and provide quantitative estimates that can inform individualized risk-benefit discussions in daily practice.
